# Age at menarche in Canada: results from the National Longitudinal Survey of Children & Youth

**DOI:** 10.1186/1471-2458-10-736

**Published:** 2010-11-28

**Authors:** Ban Al-Sahab, Chris I Ardern, Mazen J Hamadeh, Hala Tamim

**Affiliations:** 1 Kinesiology & Health Science, York University, Ontario, Canada

## Abstract

**Background:**

Given the downward trend in age at menarche and its implications for the reproductive health and wellbeing of women, little is known about menarcheal age in Canada. Most Canadian studies are only representative of specific populations. The present study, therefore, aims to assess the distribution of age at menarche for Canadian girls and explore its variation across socio-economic and demographic factors.

**Methods:**

The analysis of the study was based on all female respondents aged 14 to 17 years during Cycle 4 (2000/2001) of the National Longitudinal Survey of Children & Youth (NLSCY). The main outcome was age at menarche assessed as the month and year of the occurrence of the first menstrual cycle. Kaplan Meier was used to estimate the mean and median of age at menarche. Chi-square test was used to assess the differences in early, average and later maturers across the different levels of socio-economic and demographic variables. Bootstrapping was performed to account for the complex sampling design.

**Results:**

The total number of girls analyzed in this study was 1,403 weighted to represent 601,911 Canadian girls. The estimated mean and median of age at menarche was 12.72 years (standard deviation = 1.05) and 12.67 years, respectively. The proportions of early (< 11.53 years), average (≥11.53 years and ≤13.91 years) and late maturers (> 13.91 years) were 14.6% (95% confidence interval (CI): 11.92-17.35), 68.0% (95% CI: 63.82-72.17) and 17.4% (95% CI: 14.10-20.63), respectively. Variations across the menarcheal groups were statistically significant for the province of residence, household income and family type.

**Conclusion:**

The findings of the study pave the way for future Canadian research. More studies are warranted to understand menarcheal age in terms of its variation across the provinces, the secular trend over time and its potential predictors.

## Background

Menarche, the first menstrual period, is a critical biomarker in the reproductive life of females [1,2]. It serves as an intermediate health outcome that affects the women's wellbeing at later stages of life [3]. Although research usually overlooks intermediate health outcomes [3], age at menarche is gaining more attention as a considerable body of evidence shows that it has declined in the past century [3,4].

Early menarche is among the few established risk factors for breast cancer [5,6]. It has also been associated with the metabolic syndrome [7] and overweight [8,9]. Indirectly, it also poses a public health concern as it may result in earlier onset of sexual activity [10]. Depression, eating disorders and poor school performance are among the other teenage problems that have been associated with early menarche [11].

The first scientific records on age at menarche are 150 years old [12]. The menarcheal age in the mid 19^th ^century ranged from 16-17 years of age [12]. More recently and based on studies from 67 countries published between the 1960 s and the 1990 s, the mean age at menarche was reported to be 13.53 years (standard deviation ± 0.98) [2]. This trend signifies a decline rate of 3 to 4 months per decade [12]. Although it has been suggested that the downward trend has slowed, or even stopped, in some European countries [12], it is still ongoing in the US and Asia [3]. A national study in the United States has shown that the age at menarche has dropped from 12.75 years in the 1960 s to 12.5 years in the 1990 s and again to 12.3 years in the 2000 s [13].

Given the downward trend in age at menarche and its implications for the reproductive health and wellbeing of women, little is known about menarcheal age in Canada. Most Canadian studies are only representative of specific populations [14-19]. In 1992-1996, a cohort study in Ontario reported the median age at menarche to be 13.6 years [14], while it was found to be 12.1 years in Quebec City in 1986-1988 [15]. Only one national study, using cross-sectional data from the Canadian Community Health Survey (2004), assessed the trend in age at menarche among Canadian women 15 years of age and older with years of birth ranging from 1930 s to 2000 s [20]. Although the study was highly subject to recall bias as the accuracy of reporting age at menarche decreases with an increase in recall interval [21], nevertheless, the measured mean age at menarche between the 1930 s and 2000 s was reported to be 12.9 years [20]. The present study, however, is the first nationwide study that examined menarcheal timing among adolescent girls across the provinces in Canada. Using the National Longitudinal Survey of Children and Youth (NLSCY), the study assesses the distribution of age at menarche for Canadian girls (14-17 years old) in 2000/2001 and explores its variation across socio-economic and demographic factors.

## Methods

The analysis of this study was based on the "National Longitudinal Survey of Children and Youth" (NLSCY). The NLSCY study was initiated in 1994 by Statistics Canada and Human Resources and Social Development Canada. It aimed at assessing the development, health and wellbeing of Canadian children and youth. The study started with a representative sample of children aged 0 to 11 years from the 10 provinces of Canada. Since 1994, the children have been followed up through cycles of two-year intervals. At all cycles, the NLSCY administers different questionnaires for the person most knowledgeable (PMK) of the index child and/or the index child himself/herself. The NLSCY has been approved by the Chief Statistician of Statistics Canada, after going through various stages of internal reviews by advisory experts and field testing. Further information on NLSCY is available in other references [22].

Participants of the present study were female respondents aged between 14 and 17 years of age at cycle 4 (2000/2001). The main outcome was age at menarche assessed as the month and year of the occurrence of the first menstrual cycle. It was self reported by the index child in the "self-complete questionnaires"; whereby the child completes the questionnaire by themselves and places it in a sealed envelope to ensure confidentiality. Age at menarche was categorised into three levels: early, average and late menarche. The age limit for early and late menarche was determined as approximately 1 standard deviation (SD) away from the estimated mean age at menarche [3,4]. Premenarcheal girls were classified in the late menarcheal group [3].

The main independent variables were socio-economic factors, in particular, household income, PMK education, place of residence and PMK employment status in the past 12 months; as well as, demographic factors, specifically, province of residence, PMK immigration status, ethnicity, place of birth, language first learned at home and family type. Household income was categorized by Statistics Canada into 5 levels (lowest, lower middle, middle, upper middle and highest) based on the household size and the total household income from all sources in the past 12 months. Family type was defined as whether the child was living with both of his/her parents (biological, step or foster parents). All variables were reported by the PMK of the index child at cycle 4.

Kaplan Meier analysis employing population weights was used to estimate the mean, median and standard deviation (SD) of age at menarche. Accordingly, the distribution of early, average and late menarche was calculated. The differences in the distribution of age at menarche across the different levels of each of the independent variables were tested using chi-square tests by applying normalized weights. To account for the complex sampling design, bootstrapping was performed. Population weights, normalized weights and bootstrap weights were all created by Statistics Canada and provided with the NLSCY data file. All analyses, in exception to bootstrapping, were conducted using the Statistical Package for Social Sciences (SPSS, version 17.0). Bootstrapping was performed using the Statistical Analysis Software (SAS, version 9.2). Statistical significance for all analyses was set at alpha <0.05.

## Results

**Figure 1:**
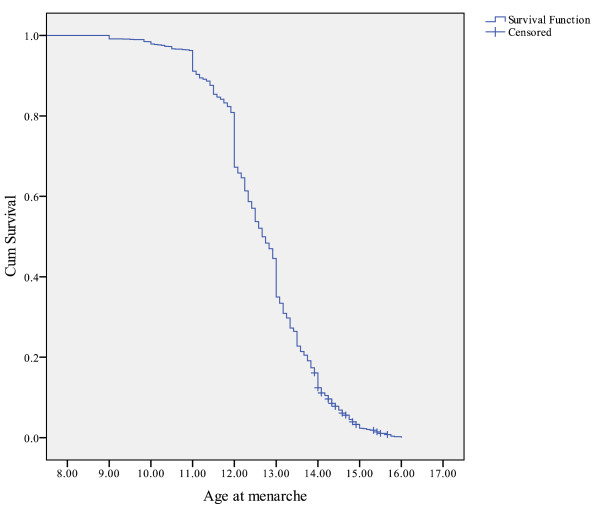
**Kaplan-Meier survival probability of attaining menarche among Canadian girls (2000/2001)**.

Around 24.3% of girls aged 14 to 17 years at cycle 4 had missing information for age at menarche. Therefore, the total number of girls analyzed in this study was 1,403 weighted to represent 601,911 Canadian girls. In 92.1% (95% confidence interval (CI): 90.2-93.9) of the study participants, the PMK was the mother while 6.7% (95% CI: 5.1-8.3) of the PMKs were fathers. The vast majority of the girls (97.3%, 95% CI: 95.84-98.86) had started menstruating at the time of cycle 4. The mean age of premenarcheal girls at the time of interview was 14.58 years (SD = 0.49). Using Kaplan Meier analysis, the estimated mean and median of age at menarche was 12.72 years (SD = 1.05) and 12.67 years, respectively. Figure 1 represents the survival probability of attaining menarche across the adolescence years. Half of the girls attained their menarche between the ages of 12.00 and 13.50 years. The proportions of early (< 11.53 years), average (≥11.53 years and ≤13.91 years) and late maturers (> 13.91 years) were 14.6% (95% CI: 11.92-17.35), 68.0% (95% CI: 63.82-72.17) and 17.4% (95% CI: 14.10-20.63), respectively.

**Figure 2:**
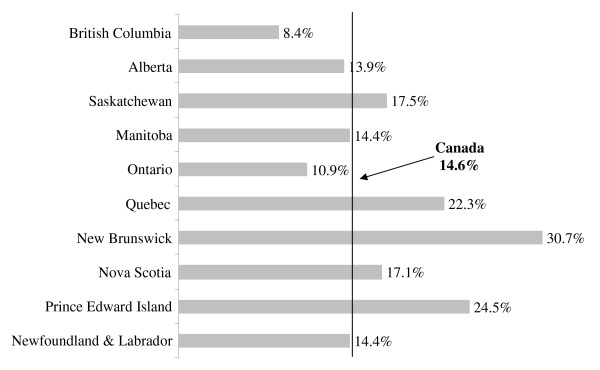
**Distribution of early menarche rates across the Canadian provinces (2000/01)**.

**Figure 3:**
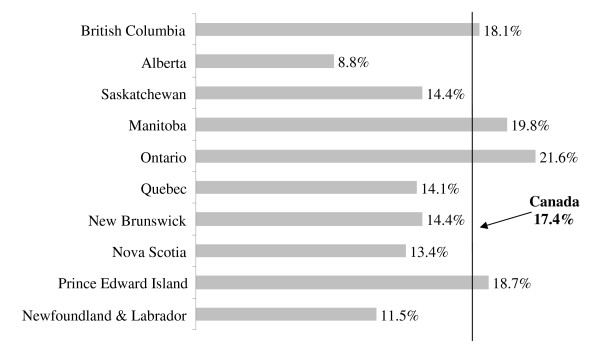
**Distribution of late menarche rates across the Canadian provinces (2000/01)**.

The distribution of early and late menarche across the Canadian provinces is illustrated in Figure 2 &3. The rates of early and late menarche in the Canadian provinces were relatively comparable to the national average. British Columbia, however, had the lowest rate of early menarche (8.4%) while New Brunswick (30.7%), Prince Edward Island (24.5%) and Quebec (22.3%) had the highest rates. On the other hand, Alberta (8.8%) had the lowest rate of late menarche and Ontario (21.6%) had the highest proportion of late maturing girls. The variation across the provinces was statistically significant (p-value= 0.013) as presented in Table 1. Other than the province of residence, income and living with both parents was found to be associated with age at menarche. The relationship between income and age at menarche was different for early and late maturers. While income increased, the proportion of early maturing girls decreased but the proportion of late maturers increased. Moreover, girls living with both parents had higher percentage of attaining late menarche than early menarche. No other socio-economic or demographic variables were found to be associated with age at menarche.

**Table 1:** Distribution of socio-economic and demographic characteristics of Canadian girls by age at menarche

	Age at menarche				
	Sample size N*	Early N*(%)^**†**^	Average N*(%)^**†**^	Late N*(%)^**†**^	P Value
***Socio-economic Variables***					
Household income					**0.018**
Lowest & lower middle	88	19 (21.2)	67 (76.0)	2 (2.8)	
Middle	347	73 (21.1)	214 (61.7)	59 (17.1)	
Upper middle	475	62 (13.1)	333 (70.0)	80 (16.9)	
Highest	487	50 (10.3)	336 (68.9)	101 (20.8)	
PMK Education					0.495
Less than Secondary	167	34 (20.1)	94 (56.5)	39 (23.4)	
Secondary school graduation	340	46 (13.4)	236 (69.3)	59 (17.3)	
Beyond high school	311	38 (12.1)	226 (72.5)	48 (15.4)	
College or university degree (including trade)	558	78 (14.0)	382 (68.4)	98 (17.6)	
Place of residence					0.685
Rural area	195	27 (13.9)	140 (71.8)	28 (14.2)	
Urban, population ≤499,999	668	89 (13.3)	462 (69.2)	117 (17.5)	
Urban, population ≥ 500,000	540	89 (16.5)	352 (65.2)	99 (18.3)	
PMK working status in the past 12 months					0.098
No	203	47 (23.2)	121 (59.7)	35 (17.2)	
Yes	1174	151 (12.9)	814 (69.3)	209 (17.8)	
***Demographic Variables***					
Province					**0.013**
Newfoundland & Labrador	31	5 (14.4)	23 (74.1)	4 (11.5)	
Prince Edward Island	8	2 (24.5)	4 (56.8)	1 (18.7)	
Nova Scotia	43	7 (17.1)	30 (69.5)	6 (13.4)	
New Brunswick	34	11 (30.7)	19 (54.9)	5 (14.4)	
Quebec	324	72 (22.3)	206 (63.6)	46 (14.1)	
Ontario	561	61 (10.9)	379 (67.5)	121 (21.6)	
Manitoba	52	8 (14.4)	34 (65.8)	10 (19.8)	
Saskatchewan	48	8 (17.5)	33 (68.1)	7 (14.4)	
Alberta	116	16 (13.9)	89 (77.3)	10 (8.8)	
British Columbia	185	16 (8.4)	136 (73.5)	33 (18.1)	
PMK Immigration status					0.634
No	1159	155 (13.4)	796 (68.7)	207 (17.9)	
Yes	210	38 (17.9)	136 (64.8)	36 (17.3)	
Place of birth					0.063
Others	60	23 (38.3)	18 (29.8)	19 (31.9)	
Canada	1325	172 (13.0)	929 (70.1)	224 (16.9)	
Ethnicity					0.079
Non-white	111	31 (27.7)	71 (64.1)	9 (8.2)	
White	1241	168 (13.5)	850 (68.5)	223 (18.0)	
Language first learnt at home					0.164
French and/or English	1307	169 (12.9)	906 (69.3)	233 (17.8)	
Other	70	23 (32.1)	37 (52.3)	11 (15.6)	
Living with both parents					**0.020**
No	331	74 (22.5)	217 (65.6)	39 (11.9)	
Yes	1072	131 (12.2)	737 (68.7)	204 (19.1)	

* Sample size is estimated using normalized weights

^† ^Frequencies are row percentages estimated using normalized weights.

## Discussion

To our knowledge, the present study is among the first to explore the national distribution of age at menarche among adolescent girls in Canada and explore its variation across socio-economic and demographic variables. The mean age at menarche in this study was estimated to be 12.72 years (SD = 1.05). Almost 14.6% of the girls attained early menarche, while 17.4% were late maturers. Variations across the menarcheal groups were statistically significant for the province of residence, household income and family type.

The study's mean age at menarche is slightly higher than the age reported in the United States (US). In 1999-2002, the national age at menarche in the US was estimated to be 12.34 years (95% CI = 12.24 to 12.45 years) [13]. Compared to the literature published between 1995 to 1998 from other developed countries, the average Canadian age at menarche is lower than the average age reported in Australia (13.0), Russia (13.0) and Norway (13.2) [2]. Nationally, only one previous study by Harris et al. (2008) assessed the age at menarche among Canadian women of all ages [20]. Due to the wide age range of the sampled women and the sampling period of 70 years, the average age at menarche does not represent the current status quo of menarche in Canada. It was measured as 12.9 years for the time period between the 1930 s and 2000 s. Compared to the present study, the average age at menarche reported by Harris et al. (2008) was slightly higher [20]. Although it might not be appropriate to compare both studies, this difference may be attributed to recall bias or an actual decline in menarcheal timing.

In the present study, there were significant provincial variations in the rates of early, average and late menarche groups. New Brunswick, Prince Edward Island and Quebec had the highest proportions of early menarche girls while Ontario had the highest proportions of late maturers. In the Canadian literature, only studies from Quebec and Ontario have previously reported menarcheal timing. In Quebec City, during the late 1980 s, the mean age at menarche was measured to be 11.4 years [23]; much lower than the national averages calculated by Harris et al. (2008) and the present study. This low mean is in agreement with the high early menarche rates in Quebec observed in the present study. On the other hand, a study in Ontario (1992-1996) reported the median age at menarche as 13.6 years [14]. This elevated median also supports the present findings of having higher rates of late maturers in Ontario. Given the lack of studies on age at menarche in Canada, little is known about the causes leading to these variations. It can be speculated that the diversity in the ethnic backgrounds of the provinces' residents, differences in lifestyles, and disparity in socio-economic classes might attribute to these provincial variations. More studies, however, are warranted to understand the underlying causes for this phenomenon. Nevertheless, by looking at the immigrant composition of the Canadian provinces, the highest proportion of foreign-born population in 2001 as reported by Statistics Canada is located in Ontario (26.8%), British Columbia (26.1%), Alberta (14.9%) and Manitoba (12.1%) [24]. Interestingly, all of these provinces reported lower prevalence rate of early menarche than the national average. On the other hand, New Brunswick and Prince Edward Island who had the highest early menarche rates had low foreign-born population (3.1% for both) [24].

Out of all the socio-economic indicators, only income was found to have a significant effect on age at menarche. High income was associated with lower early menarche rates but higher rates for late menarche. The study findings are in disagreement with the literature, whereby low socio-economic status has been found to delay menarcheal timing [12]. As explained in earlier studies, higher socio-economic status improves nutrition and consequently favours early menarche [2,12]. This is further justified by Frisch & McArthur theory (1974) that states a critical proportion of body fat (17%-22%) may be required to trigger menarche [25]. Despite the above, one Canadian study that measured the pubertal developmental scale among boys and girls had findings similar to the present study [26]. It revealed that low family income predicts the young initiation of pubertal development [26]. A possible explanation for this relationship is that high socio-economic status diminishes associated stress levels and hence delays menarche. Another possible explanation can be attributed to the fact that obesity predicts earlier menarche [1,4] and obesity in Canada is found to be more prevalent among lower socio-economic status groups [27,28]. In the Canadian adult population, women but not men of lower income quintiles were more likely to be obese than those with higher income quintiles [27]. In the adolescent population, children of middle-income families were more likely to be obese than their counterparts. The trend however was more pronounced by level of education; whereby adolescents living in households with no members having high school diploma were more prone to be overweight and obese than those who had a household member with post-secondary education [28]. Although not significant, PMK working status in the past 12 months in the present study also showed a trend of higher early menarche rates among unemployed PMKs.

In accordance with the literature, living in a signal parent family increased the likelihood of attaining early menarche [29,30]. Roman et al. (2003) showed that girls without fathers in the home are 2.62 times (95% CI: 1.58-4.33) more like to have menarche before the age of 12 years [30]. Moreover, a national study in the United Kingdom also revealed that absence of a father predicts earlier menarche [29]. Belsky et al. (1991) hypothesized that the physical and psychosocial stress caused by such environmental cues induces metabolic changes that promotes young menarcheal timing [31].

Ethnicity and place of birth, in the present study, approached significance with age at menarche (p-value is 0.079 and 0.063, respectively). Based on the literature, racial differences are evident especially between white and black girls [13]. On average, black girls attain menarche earlier than white girls [13]. A national study in the United States (1998-1994) revealed the average age at menarche for white girls was 12.60 years (95% CI: 12.48-12.71), whereas it was 12.14 years (95% CI: 11.87-12.39) for black girls [32]. The reason for this difference is unclear but can be due to genetics and/or nutrition [11]. Difference in the adiposity level between white and black girls at this age might be another explanation. Similar trend was observed in this study, where non-white girls had a higher proportion of early maturers.

Several limitations of the current study warrant mention. A total of 24.3% of cycle 4 respondents were excluded from the analysis due to missing information on age at menarche. No differences across socio-economic and demographic characteristics, except for the province of residence, were evident between girls included in the analysis and those excluded. Girls with missing data were slightly more likely to be residents of the Eastern provinces. Furthermore, the study would have greatly benefited by analyzing data on the predictors of age at menarche. Unfortunately, the data on predictors is not available for cycle 4. Prenatal factors, among the important correlates of early menarche, are missing for this age group [1,3,4]. No information, also, has been collected on maternal age at menarche; which is considered the most important predictor of menarche [33]. Finally, the study results are reflective of the years 2000/2001. Although more recent cycles of the NSCLY are available for the year 2006/2007, generalizability of such results would be limited to the original NLSCY cohort that was sampled in 1994. At each cycle, Statistics Canada creates cross-sectional weights to account for demographic changes in the population. These weights allow the generalizability of the results to the Canadian population. No cross-sectional weights, however, were created after cycle 4. Despite the above, the study had several advantages. Menarche data were collected close to the time of the event, reducing the likelihood of recall bias. Moreover, the use of survival analysis and bootstrapping techniques improved the accuracy of the results.

## Conclusions

To our knowledge, this is the first study to explore the national distribution of age at menarche among adolescent girls in Canada and explore its variation across socio-economic and demographic variables. The findings of the study pave the way for future Canadian research. More studies are warranted to understand menarcheal age in terms of its variation across the provinces, the secular trend over time and its potential predictors. Future studies should, as well, explore the effect of living with a single parent on age at menarche.

## Abbreviations

CI: Confidence interval; NLSCY: National Longitudinal Survey of Children & Youth; OR: Odds ratio; PMK: Person most knowledgeable; SAS: Statistical Analysis Software; SD: Standard deviation; SPSS: Statistical Package for Social Sciences; US: United States

## Competing interests

The authors declare that they have no competing interests.

## Authors' contributions

BAS: Generated the idea and the design of the study; performed analysis and write up of the paper. CIA: Made substantial contributions to the design and interpretation of the results; provided technical support and advice; reviewed the final paper and all its drafts. MJH: Made substantial contributions to the design and interpretation of the results; provided technical support and advice; reviewed the final paper and all its drafts. HT: Generating the idea and design of the study; supervised the analysis, interpretation of the results and write up of the manuscript; reviewed the final paper and all its drafts.

All authors read and approved the final manuscript.

## Pre-publication history

The pre-publication history for this paper can be accessed here:

http://www.biomedcentral.com/1471-2458/10/736/prepub
